# New Insights into Behçet's Syndrome Metabolic Reprogramming: Citrate Pathway Dysregulation

**DOI:** 10.1155/2018/1419352

**Published:** 2018-06-28

**Authors:** Anna Santarsiero, Pietro Leccese, Paolo Convertini, Angela Padula, Paolo Abriola, Salvatore D'Angelo, Faustino Bisaccia, Vittoria Infantino

**Affiliations:** ^1^Department of Science, University of Basilicata, Potenza, Italy; ^2^Rheumatology Department of Lucania, Rheumatology Institute of Lucania (IReL), San Carlo Hospital of Potenza and Madonna delle Grazie Hospital of Matera, Potenza, Italy; ^3^Basilicata Ricerca Biomedica (BRB) Foundation, Potenza, Italy

## Abstract

To date, a major research effort on Behçet's syndrome (BS) has been concentrated on immunological aspects. Little is known about the metabolic reprogramming in BS. Citrate is an intermediary metabolite synthesized in mitochondria, and when transported into the cytosol by the mitochondrial citrate carrier—SLC25A1-encoded protein—it is cleaved into acetyl-CoA and oxaloacetate by ATP citrate lyase (ACLY). In induced macrophages, mitochondrial citrate is necessary for the production of inflammatory mediators. The aim of our study was to evaluate SLC25A1 and ACLY expression levels in BS patients. Following a power analysis undertaken on few random samples, the number of enrolled patients was set. Thirty-nine consecutive BS patients fulfilling ISG criteria, and 21 healthy controls suitable for age and sex were recruited. BS patients were divided into two groups according to the presence (active) or absence (inactive) of clinical manifestations. Real-time PCR experiments were performed on PBMCs to quantify SLC25A1 and ACLY mRNA levels. Data processing through the Kruskal-Wallis test and Dunn's multiple comparison test as post hoc showed higher SLC25A1 and ACLY mRNA levels in BS patients compared to those in healthy controls. Therefore, SLC25A1 and ACLY upregulation suggests that metabolic reprogramming in BS involves the citrate pathway dysregulation.

## 1. Introduction

Behçet's syndrome (BS) is a multisystemic inflammatory disorder classified among primary vasculitis. The diagnosis is based on clinical signs due to the lack of specific tests. Typical clinical manifestations include oral and genital ulcers, uveitis, and skin lesions. The involvement of the central nervous system, vessel tree, and gastrointestinal tract even if less frequent can be life-threatening. The disease is characterized by a relapsing-remitting course with a period of substantial wellbeing between the phases [1]. BS is more common in certain geographic regions, and in nonendemic areas, the disease tends to be more frequent in female with a less severe course [2]. The pathogenesis of BS is still unknown. However, over the past years, genetic and immunological mechanisms have been investigated [3]. Little is known about metabolic signals which can be responsible for a metabolic reprogramming linked to the induction and/or control of inflammation in BS. Belguendouz et al. analyzed the arginine metabolism in BS patients. They found that nitric oxide and urea production were significantly increased in patients with active BS and NOS/arginase balance resulted depending on clinical expression [4].

Citrate is a key metabolite for energy production. Citrate is synthesized by citrate synthase from acetyl-CoA and oxaloacetate (OAA) in the mitochondrion where it usually enters the Krebs cycle and promotes oxidative phosphorylation.

Immune response of activated macrophages and dendritic cells requires a metabolic reprogramming during which citrate is diverted away from the Krebs cycle toward the cytosol where it can be used to trigger several cellular functions [5]. Part of the mitochondrial citrate is also converted in itaconate which acts as a bactericide and negative regulator of the inflammation [6, 7].

Citrate exported to the cytosol by means of the mitochondrial citrate carrier (CIC)—SLC25A1-encoded protein—is cleaved by ATP citrate lyase (ACLY) to acetyl-coenzyme A (acetyl-CoA) and OAA [8]. Acetyl-CoA is the precursor for fatty acid and sterol biosynthesis and the universal donor for acetylation reactions. OAA is reduced to malate by cytosolic malate dehydrogenase and converted to pyruvate via malic enzyme with generation of cytosolic NADPH plus H^+^ (necessary for fatty acid and sterol synthesis). We refer to CIC plus ACLY as a “citrate pathway” [8].

Recent studies have shown that the citrate pathway is activated in LPS- and cytokine-triggered macrophages [5, 9]. Indeed, an early upregulation of ACLY gene followed by an increase in SCL25A1 expression levels has been found [10–12]. Moreover, both endogenous and exogenous inducers produce an increase in cytosolic citrate levels in macrophages. Of note, acetyl-CoA- and OAA citrate-derived metabolites are essential to synthesize inflammatory mediators. Acetyl-CoA provides units for lipid elongation, including arachidonic acid, which is needed for the production of prostaglandins. OAA leads to NADPH production, necessary for NADPH oxidase and inducible NO synthase (iNOS) to generate ROS and NO, respectively. Indeed, CIC or ACLY activity inhibition by gene silencing as well as by specific inhibitors reduces the amount of ROS, NO, and proinflammatory prostaglandins [8, 10–12]. Furthermore, the addition of exogenous acetate nearly entirely prevented the inhibition of PGE_2_ synthesis by citrate export pathway-specific inhibitors [11].

Thus, citrate diversion from the Krebs cycle to the citrate export pathway guarantees a strong inflammatory response and induces a Krebs cycle breaking point downstream of citrate. Isocitrate dehydrogenase (IDH) profound downregulation described in LPS-activated macrophages by Jha et al. [13] supports these metabolic changes and highlights the role of SLC25A1 and ACLY in inflammation.

Of note, increased levels of SLC25A1 and ACLY mRNAs have also been observed in activated natural killer (NK) cells [14], lymphocytes actively involved in chronic inflammatory diseases, and in obesity which is often associated to a chronic low-grade inflammation [15]. ACLY and SLC25A1 upregulation in induced NK cells is mediated by the sterol regulatory element-binding protein (SREBP) [14], according to the presence of sterol regulatory elements (SREs) in both ACLY and SLC25A1 gene promoters [16, 17]. Furthermore, Assmann et al. demonstrate that ACLY and CIC, as part of the citrate-malate shuttle, provide electron transfer from cytosolic NADH to mitochondrial NADH to sustain OXPHOS and at the same time to regenerate cytosolic NAD+ [14]. The citrate-malate shuttle activation is one of the metabolic changes ensuring an effective NK cell response during inflammation.

SLC25A1 and ACLY upregulation together with the citrate accumulation occurring in immune cells points out the importance of the breakpoint in the Krebs cycle downstream of citrate [18].

Taken together, these findings provide evidence that the citrate export pathway, via CIC and ACLY, has an essential function in metabolic reprogramming of immune cells and shed light on the relationship between energy metabolism and inflammatory diseases.

As BS displays inflammatory features and almost nothing is known about metabolic reprogramming linked to the disease, in our study, we analyzed the expression pattern of SLC25A1 and ACLY genes in BS patients and their correlation with the disease activity.

## 2. Materials and Methods

### 2.1. Study Population

Thirty-nine consecutive BS patients, fulfilling the International Study Group (ISG) classification criteria, followed at the outpatient clinic of Rheumatology Department of Lucania, and 21 matched healthy volunteers were enrolled. Healthy controls were recruited among hospital workers and were unrelated to each other or to the BS patients. All subjects signed written informed consent before participation. Research was carried out under the institutionally approved internal review board protocol and in accordance with the Declaration of Helsinki. For all subjects, the following data were collected: age, sex, erythrocyte sedimentation rate (ESR), and C-reactive protein (CRP) values. All BS patients were evaluated by the same rheumatologist, and an ocular examination was performed by an ophthalmologist. Based on the presence or not of at least one clinical manifestation, BS patients were divided in two groups: active and inactive. Disease activity was assessed by BDCAF (Behçet's Disease Current Activity Form) and BSAS (Behçet's Syndrome Activity Score) score systems. In the BS group, disease duration was calculated as the time elapsed since the onset of first clinical manifestation.

### 2.2. Determination of IL-1*β* levels in Serum

Venous blood was drawn from each subject enrolled. After clotting, serum was obtained by centrifugation at 2000 ×g for 10 minutes and stored at −80°C and thawed immediately before use. Serum samples were centrifuged at 9000 ×g for 5 minutes at 4°C and subjected to ELISA (IL-1*β*; ImmunoTools, Friesoythe, Germany). Each sample was tested in duplicate according to the manufacturer's procedure. In brief, microtiter ELISA plate was coated with human monoclonal antibody against IL-1*β*. Standards and serum samples were added to the wells and incubated for 2 hours at 37°C. After washing five times, incubation with biotinylated anti-human IL-1*β* antibody was performed for 2 hours at room temperature. Following a second wash, 100 *μ*L of streptavidin-horseradish peroxidase was put into the wells and incubated for 30 minutes. Color development was ensured by using tetramethylbenzidine substrate solution. The reaction was stopped by the addition of 50 *μ*L of 2 M of sulfuric acid. Finally, optical density at 450 nm was measured by GloMax®-Multi Detection System (Promega, Madison, WI). The standard curve was prepared on the basis of seven IL-1*β* dilutions. The detection range was 23–1500 pg/mL.

### 2.3. Isolation of PBMCs from Whole Blood

Peripheral blood mononuclear cells (PBMCs) were isolated from heparinized blood using Histopaque-1077 (Sigma-Aldrich, St Louis, MO) density centrifugation. Whole blood was mixed with HBSS (Hanks' balanced salt solution, Gibco, Grand Island, NY) at a ratio of 1 : 2 (*v*/*v*), layered on the top of Histopaque-1077 and centrifuged at 1000 ×g at room temperature for 15 minutes. The mononuclear cell layer was recovered and washed twice in HBSS for 10 minutes. Cell counting was done with the automated handheld Scepter 2.0 Cell Counter (Merck Millipore, Switzerland).

### 2.4. Quantitative Real-Time PCR

Total RNA was extracted from 2 × 10^6^ PBMCs using the RNeasy Plus Mini Kit (Qiagen, Hilden, Germany) as per the manufacturer's instructions. Complementary DNA was synthesized by GeneAmp™ RNA PCR Core Kit (Thermo Fisher Scientific, Grand Island, NY) with random hexamers and murine leukemia virus reverse transcriptase (15 minutes at 42°C and 5 minutes at 99°C). Real-time PCR experiments were performed in triplicate on the 7500 Fast Real-Time PCR System (Thermo Fisher Scientific) with SLC25A1 (Hs01105608, amplicon length 75 bp, RefSeq NM_001256534.1—exon boundary 2-3), ACLY (Hs00982738, amplicon length 69 bp, RefSeq NM_001096.2—exon boundary 28-29), and *β*-actin (Hs01060665, amplicon length 63 bp, RefSeq NM_001101.3—exon boundary 2-3) TaqMan Gene Expression Assays (Thermo Fisher Scientific). *β*-Actin was used as endogenous reference gene for normalization of SLC25A1 and ACLY expression levels. To this end, the *β*-actin Ct value was subtracted from the target gene Ct value, generating a ΔCt value. ΔΔCt was calculated by subtracting the mean value of ΔCt of the control group from ΔCt of each patient. Finally, fold changes in SLC25A1 and ACLY gene expression were calculated via the comparative 2^−ΔΔCt^ method using the formula: 2^−ΔΔCt^ = ΔCt_BS patient_ − ΔCt_control_.

### 2.5. Statistical Analysis

After a preliminary study on 5 active and 5 inactive BS patients, a power analysis was performed to determine the sample size. The research was designed using mean values and pooled standard deviations of SLC25A1 and ACLY mRNA levels from the aforementioned few subjects to have a statistical power of 80% with an *α*-level of 0.05. The Shapiro-Wilk test at an *α*-level of 0.05 was used to assess for data normality. Statistical significance of differences of SLC25A1 and ACLY expression levels was determined by using the Kruskal-Wallis test followed by the Dunn's multiple comparison test with *p* values adjusted with the Benjamini-Hochberg method. Differences were considered as significant (^∗^*p* < 0.05), very significant (^∗∗^*p* < 0.01), and highly significant (^∗∗∗^*p* < 0.001). All statistical analyses were performed by using RStudio (version 1.1.423, RStudio Inc., Boston, MA).

## 3. Results

### 3.1. Sample Size Estimation

Preliminary data on 5 active and 5 inactive BS patients indicated that both SLC25A1 and ACLY were upregulated during the active phase. In particular, the differences of mean values between mRNAs from active and inactive BS patients were 3.64 for SLC25A1 and 1.00 for ACLY. The pooled standard deviations were 3.47 and 1.03 for SLC25A1 and ACLY, respectively. A power analysis by using preliminary data was performed to fix the sample size in order to obtain 80% power to detect the above reported differences at the *α*-level of 0.05. We achieved that active and inactive BS groups would need to consist of 16 and 18 patients for SLC25A1 and ACLY, respectively. We recruited 18 active and 21 inactive BS patients.

### 3.2. Demographic and Clinical Features of the Patients

Among the 39 consecutive BS patients enrolled, all received a pharmacological treatment, 18 had active disease (mean ± SD age = 41.3 ± 11.3 years) and the remaining 21 had no clinical manifestation (mean ± SD age = 42.8 ± 13.1 years). The male : female ratio in both groups was 2 : 1. The healthy controls recruited were suitable for sex and age (M : F = 14 : 7, mean ± SD age = 38.1 ± 12.1 years). The mean disease duration was 22.8 ± 9.7 years for the active group and 21.4 ± 11.4 years for the inactive BS group. ESR and CRP values were similar among the three groups (Table 1). On the contrary, increased serum levels of IL-1*β* were found in active BS patients (mean ± SD: 463.1 ± 85.9 pg/mL) compared to the inactive BS patients (mean ± SD: 393.8 ± 124.9 pg/mL) and healthy controls (mean ± SD = 234.4 ± 123.2 pg/mL, *p* value = 0.016, Kruskal-Wallis test) (Table 1). BSAS and BDCAF scores resulted 5.5 and 4.5 higher in active subjects compared to those in inactive subjects, respectively. One half of the active BS patients had only one clinical manifestation. The most frequent clinical manifestation was the posterior uveitis (38.9%) present in 13 eyes. Other clinical manifestations included oral ulcers (33.3%), skin lesions (27.8%), and peripheral arthritis (22.2%). Active central nervous system vasculitis and deep venous thrombosis were found in only one male and female, respectively. Skin lesions and articular involvement were more frequent in men than in women while oral ulcers and uveitis were equally distributed. Demographic characteristics of the patients and control group are detailed in Table 1.

### 3.3. SLC25A1 mRNA Quantification

To investigate a potential link between the citrate pathway and BS, we evaluated SLC25A1 gene expression in PBMCs from inactive and active BS patients and healthy controls.

**Figure 1:**
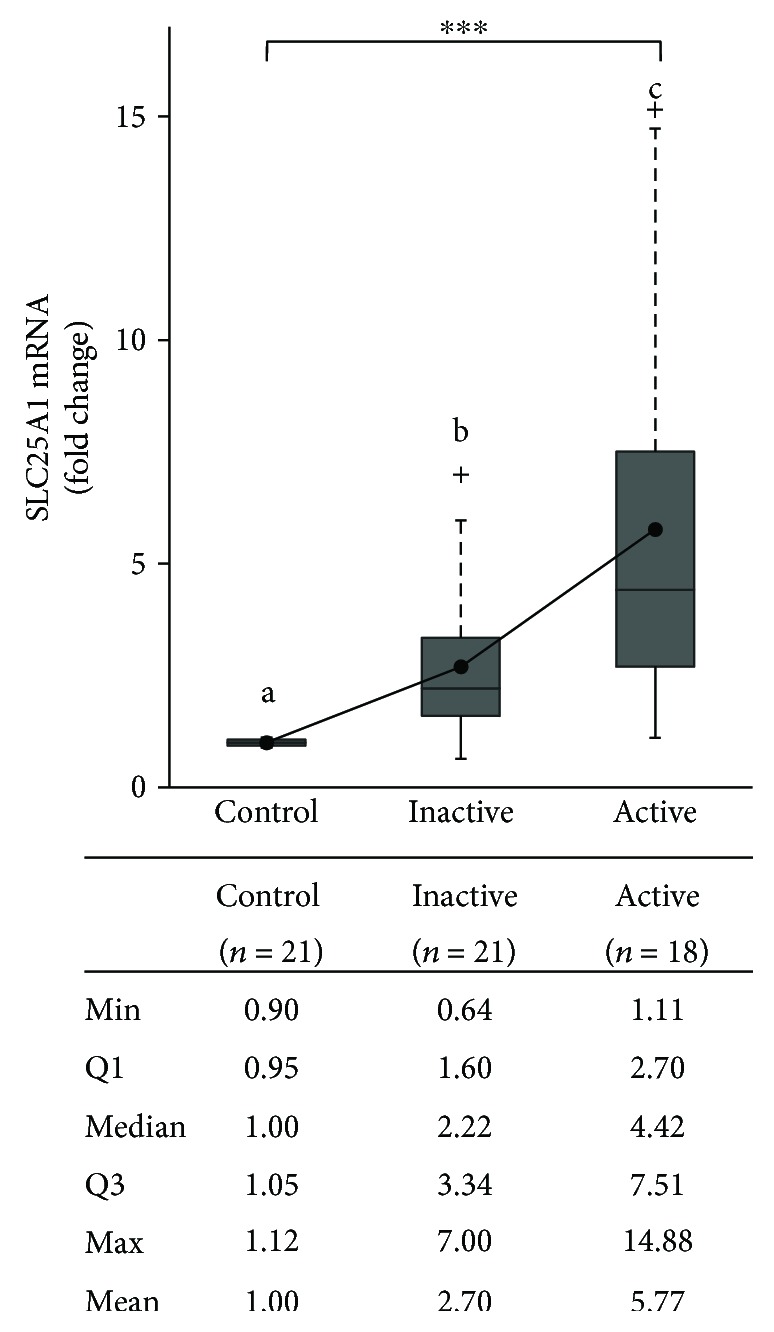
Expression levels of SLC25A1 mRNA in study population. SLC25A1 mRNA levels have been quantified in PBMCs from 21 healthy adult volunteers (control) and 39 BS patients (21 with inactive disease and 18 with active disease). Results are shown as box plots, where the horizontal line within the boxes represents the median, the boxes represent the first and third quartiles, and the bars outside the boxes represent the minimum and maximum values. Where indicated, the dashed bars represent the upper ends of the farthest observed data point within 1.5 times the interquartile range and the plus signs represent outliers. Dots within the boxes indicate the mean values. Differences among the groups were highly significant (^∗∗∗^*p* < 0.001, Kruskal-Wallis test). Pairwise comparisons were made with the post hoc Dunn's test; results are shown with letters. Distinct letters indicate that differences are significant.

SLC25A1 mRNA levels resulted higher in active BS patients (mean ± SD: 5.77 ± 4.34, median 4.42) compared to those in inactive BS patients (mean ± SD: 2.70 ± 1.83, median 2.22) and control subjects (mean ± SD: 1.00 ± 0.08) (Figure 1). The IQR (interquartile range) in active BS patients was about three-fold higher (4.81) than that in inactive BS patients (1.74). This means that 50% of active population had SLC25A1 mRNA levels from 2.7 to 7.51 while inactive patients SLC25A1 mRNA level range was from 1.6 to 3.34 (Figure 1).

To assess for normal distribution of SLC25A1 mRNA levels, we performed the Shapiro-Wilk test. While SLC25A1 expression levels were normally distributed in controls (*W* = 0.91, *p* value = 0.20), they did not follow normal distribution in inactive (*W* = 0.86, *p* value = 0.007) and active (*W* = 0.87, *p* value = 0.017) BS patients. Therefore, by using a nonparametric test, we observed statistically significant differences in SLC25A1 mRNA levels among controls and inactive and active BS patients (*χ*^2^ = 25.92, df = 2, *p* value = 2.36 × 10^−6^, Kruskal-Wallis test). Since a post hoc analysis was necessary to make comparisons between each pair of groups, the Dunn's multiple comparison test was performed. Every pairwise comparison was statistically significant (Figure 1). *p* values adjusted with the Benjamini-Hochberg method were 3.20 × 10^−3^ for control versus inactive BS patients, 1.07 × 10^−6^ for control versus active BS patients, and 1.66 × 10^−2^ for inactive versus active BS patients (Figure 1). SLC25A1 expression levels did not correlate with gender or clinical manifestation (data not shown). Altogether, our results show a great increase of SLC25A1 mRNA levels in BS compared to control subjects. Of note, a further SLC25A1 upregulation is observed during the active phase of BS.

### 3.4. ACLY mRNA Quantification

SLC25A1 upregulation indicates a possible export of the citrate in BS. To strengthen this hypothesis, we quantified ACLY mRNA.

**Figure 2:**
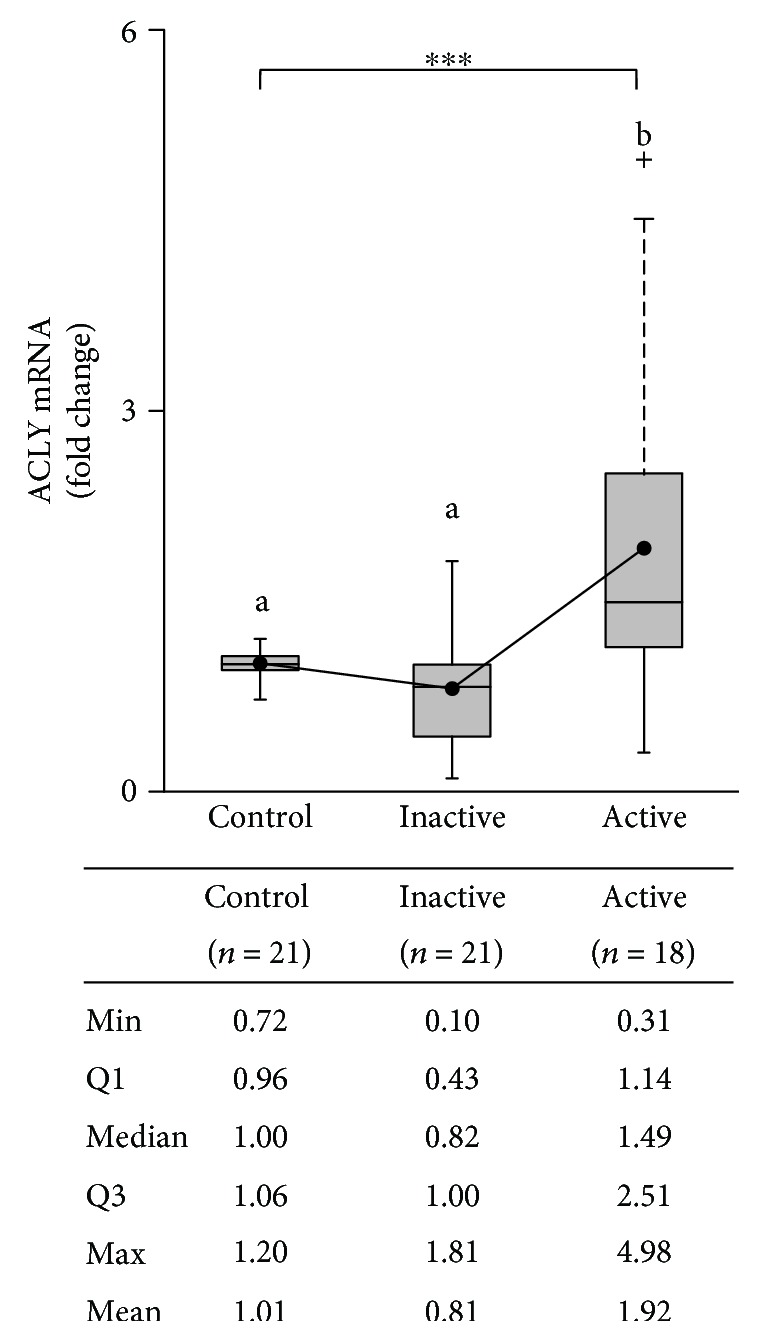
Expression levels of ACLY mRNAs in study population. ACLY mRNA levels have been quantified in PBMCs from 21 healthy adult volunteers (control) and 39 BS patients (21 with inactive disease and 18 with active disease). Results are shown as box plots, where the horizontal line within the boxes represents the median, the boxes represent the first and third quartiles, and the bars outside the boxes represent the minimum and maximum values. Where indicated, the dashed bars represent the upper ends of the farthest observed data point within 1.5 times the interquartile range and the plus signs represent outliers. Dots within the boxes indicate the mean values. Differences among the groups were highly significant (^∗∗∗^*p* < 0.001, Kruskal-Wallis test). Pairwise comparisons were made with the post hoc Dunn's test; results are shown with letters. Values sharing the same letter are not significantly different.

We found a two-fold increase of ACLY mRNA in active BS patients (mean ± SD: 1.92 ± 1.21, median 1.49) compared to controls (mean ± SD: 1.01 ± 0.14). No difference of ACLY expression levels was observed between inactive BS patients (mean ± SD: 0.81 ± 0.45, median 0.82) and the control group. It is interesting to note that ACLY mRNA levels were less than 1 for 75% of inactive patients and more than 1.14 for 75% of active patients (Figure 2). The Shapiro-Wilk test highlighted that ACLY transcript levels were normally distributed in controls (*W* = 0.92, *p* value = 0.40) and inactive BS patients (*W* = 0.97, *p* value = 0.66), instead they did not follow normal distribution in active BS patients (*W* = 0.87, *p* value = 0.019). Then, the Kruskal-Wallis test revealed statistically significant differences in ACLY mRNA levels among controls and inactive and active BS patients (*χ*^2^ = 17.75, df = 2, *p* value = 1.39 × 10^−4^) (Figure 2). The Dunn's multiple comparison test clearly indicated that differences between controls and inactive BS patients were not statistically significant (*p* value = 0.24) (Figure 2).

Notably, *p* values adjusted with the Benjamini-Hochberg method were 3.58 × 10^−2^ for control versus active BS patients and 8.48 × 10^−5^ for inactive versus active BS patients (Figure 2).

ACLY expression levels did not correlate with gender or clinical manifestation (data not shown). The results of our study indicate that ACLY gene is upregulated in active BS patients than in healthy controls. Furthermore, it has been displayed a significant increment of ACLY mRNA levels in the presence of active disease when compared to inactive disease.

## 4. Discussion

Metabolism is essential to all living cells for generating energy, signalling molecules, and synthesizing or breaking down macromolecules. Metabolic adaptation and reprogramming are frequent links between environmental changes and cell function. A metabolic reprogramming occurs concurrently during immune cell activation. In resting dendritic cells, lymphocytes, and macrophages, catabolic pathways switch to anabolic programmes after activation by various triggers. These complex events ensure that activated cells generate energy and metabolites necessary to perform their specific function. A well-known example of metabolic shift—observed in inflammatory cells such as M1 macrophages and CD4+ T helper 17 (Th17) lymphocytes—is the enhanced glycolysis (Warburg phenotype) allowing for rapid ATP production [19]. Mitochondrial Krebs cycle enzymes are also inhibited, indicating a shift of the TCA cycle from being a purely catabolic pathway generating ATP to being, at least in part, an anabolic pathway. M1 macrophages display two breaks in the Krebs cycle downstream of citrate and succinate with a downregulation of IDH and succinate dehydrogenase, respectively [5, 20]. A so rewired Krebs cycle warrants increased levels of citrate and succinate, two key metabolites crucial for immune responses. Succinate through the hypoxia-inducible factor 1 alpha (HIF1alpha) activation induces HIF1alpha target genes including IL-1*β* [21]. Citrate withdrawn by the citrate export pathway activates fatty acid synthesis (which in turn participates in prostaglandin generation) and allows for reactive nitrogen species (RNS) and ROS production. Interestingly, a citrate pathway upregulation has been found in PMBCs from children affected by Down syndrome—a genetic disorder with the occurrence of many inflammatory conditions and a permanent oxidative stress [22].

In the last years, a host of studies has demonstrated that targeting different metabolic pathways is possible to regulate immune responses and treat immune-mediated pathogenesis. Thus, immunometabolism modulation might prevent a dangerous response or provide the desired response, so becoming an attractive alternative to chemotherapy or immunosuppression.

Metabolic reprogramming of immune cells is a feature deeply associated to rheumatologic diseases such as systemic lupus erythematosus, rheumatoid arthritis, and osteoarthritis and could be a novel opportunity to manipulate cellular metabolism for therapeutic purposes [23].

BS shares clinical features with well-recognised autoinflammatory disorders. There is growing evidence that environmental factors and both viral and bacterial agents may act as BS triggers in genetically predisposed subjects; genetic factors induce the immune system hyperactivity, the expression of heat shock proteins and major histocompatibility complex (MHC) class I chain-related molecules A [24, 25].

Genome-wide association studies (GWAS) showed that several susceptibility loci explaining the genetic contribution to BS onset and development correspond to genes involved in the inflammasome pathway. The human leukocyte antigen-B51 (HLA-B∗51) is the most strongly associated risk factor for BS, but it only partially explains the genetic risk of BS [26–28]. These genes involve both innate and adaptive immunities and are shared among different immune-related disorders [24, 29].

Another point to address in clarifying the inflammatory feature of BS was related to the relationship between the excessive T cell-mediated inflammatory response and the disease activity. The helper T cell (Th) homeostasis perturbation seems to be involved in this mechanism. Higher frequency of circulating Th1/Th17 cells has been reported in active BS patients compared with inactive patients, suggesting that these cells and the interleukin 17/interleukin 23 pathway can contribute to the inflammatory reaction and have a pathogenic role in BS [30, 31]. High levels of innate immunity-related cytokines such as IL-1*β*, IL-6, IL-12, IL-23, and TNF-*α* have been found in sera of BS patients compared to those of controls [32, 33]. Among proinflammatory cytokines secreted by macrophages, IL-1*β* could play a specific role in activation and homeostasis regulation of Th cells. Interestingly, in our study, sera from active show a significant increase of IL-1*β* with respect to those from inactive BS patients and healthy controls.

Proinflammatory cytokines were also secreted by hyperactivated neutrophils, so the activation of neutrophils is another key mechanism in the pathogenesis of the disease. Previous studies reported both increased proactive neutrophils and increased generation of ROS in BS patients [34–36].

**Figure 3:**
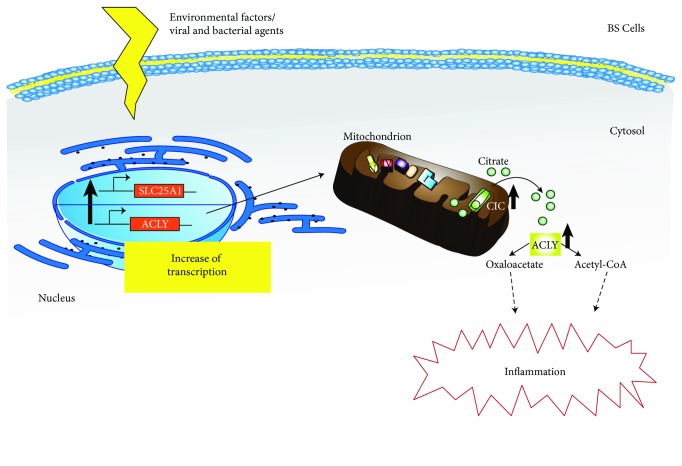
Role of the citrate pathway in Behçet's syndrome (BS). In BS cells, different triggers induce an increase of the SLC25A1 and ACLY transcription rate. The citrate carrier (CIC)—SLC25A1 encoded protein—exports citrate from mitochondria to the cytosol where it is cleaved by ACLY into oxaloacetate and acetyl-CoA. Both the metabolites support the production of the inflammatory mediators.

However, little is known about the involvement of immunometabolism in Behçet's syndrome. Here, for the first time, we have investigated the role of the citrate pathway in BS enrolling patients divided into two groups: patients having active disease and patients having no clinical manifestations. Interestingly, the presence of an active disease is linked to higher levels of SCL25A1 mRNA compared to those measured in inactive BS patients. Moreover, inactive BS patients show a significant rise of SCL25A1 mRNA with respect to control subjects, indicating a first step of upregulation related to the presence of BS and an additional one during the active phase. In light of our results, we have hypothesized that a metabolic trait of BS could be the diversion of the citrate from the Krebs cycle to the cytosol. Increased ACLY mRNA levels found in active BS patients compared to controls strengthen our idea. Therefore, SLC25A1 upregulation could allow a great export of the citrate from the mitochondria to the cytosol where it is cleaved by increased levels of ACLY thus producing acetyl-CoA and OAA. Since it has been reported that both the metabolites are used in immune cells to synthesize specific mediators of inflammation such as ROS, NO, and prostaglandins (Figure 3) [5], the increased citrate export pathway here described in BS patients could be responsible, at least in part, for the increased oxidative stress and inflammatory features of the disease.

Our results shed light on citrate pathway dysregulation occurring in BS. However, further investigations are needed to better understand its role in BS pathogenesis.

## Figures and Tables

**Table 1:** Demographic and clinical features of all subjects enrolled in the study.

Parameter	Active (*n* = 18)	Inactive (*n* = 21)	Control (*n* = 21)
Age, mean ± SD (years)	41.3 ± 11.3	42.8 ± 13.1	38.1 ± 12.1
Disease duration, mean ± SD (years)	22.8 ± 9.7	21.4 ± 11.4	
Male sex, number (%)	12 (66.7)	14 (66.7)	14 (66.7)
Female sex, number (%)	6 (33.3)	7 (33.3)	7 (33.3)
ESR (mm/1 h), mean ± SD	11.3 ± 8.2	12.9 ± 8.2	9.1 ± 6.3
CRP (mg/L), mean ± SD	1.9 ± 2.0	1.5 ± 1.9	0.8 ± 1.2
IL-1*β* (pg/mL), mean ± SD	463.1 ± 85.9	393.8 ± 124.9	234.4 ± 123.2
BSAS, mean ± SD	34.8 ± 14.7	6.1 ± 7.5	
BDCAF, mean ± SD	3.7 ± 1.7	0.8 ± 1.1	
One clinical manifestation, number (%)	9 (50.0)		
More than one clinical manifestation, number (%)	9 (50.0)		
Clinical manifestations, number (%)			
Posterior uveitis	7 (38.9)		
Oral ulcers	6 (33.3)		
Skin lesions	5 (27.8)		
Arthritis	4 (22.2)		
CNS involvement	1 (5.5)		
Vascular involvement	1 (5.5)		

All data are presented as mean ± standard deviation except gender and clinical manifestations listed as percentage. ESR = erythrocyte sedimentation rate; CRP = C-reactive protein; BSAS = Behçet's Syndrome Activity Score; BDCAF = Behçet's Disease Current Activity Form; CNS = central nervous system.

## Data Availability

The data used to support the findings of this study are available from the corresponding author upon request.
